# Revalorization of Yerba Mate Residues: Biopolymers-Based Films of Dual Wettability as Potential Mulching Materials

**DOI:** 10.3390/polym16060815

**Published:** 2024-03-14

**Authors:** Laura M. Sanchez, Jorge de Haro, Eva Domínguez, Alejandro Rodríguez, Antonio Heredia, José J. Benítez

**Affiliations:** 1 Departamento de Biología Molecular y Bioquímica, Facultad de Ciencias, Instituto de Hortofruticultura Subtropical y Mediterránea “La Mayora”, Universidad de Málaga, Consejo Superior de Investigaciones Científicas (IHSM, UMA-CSIC), 29071 Malaga, Spain; heredia@uma.es; 2 BioPrEn Group (RNM 940), Chemical Engineering Department, Instituto Químico para la Energía y el Medioambiente (IQUEMA), Faculty of Science, Universidad de Córdoba, 14014 Cordoba, Spain; q42hanij@uco.es (J.d.H.); q42ropaa@uco.es (A.R.); 3 Departamento de Mejora Genética y Biotecnología, Estación Experimental La Mayora, Algarrobo-Costa, Instituto de Hortofruticultura Subtropical y Mediterránea La Mayora, Universidad de Málaga, Consejo Superior de Investigaciones Científicas, 29750 Malaga, Spain; edominguez@eelm.csic.es; 4 Instituto de Ciencia de Materiales de Sevilla (ICMS), Centro Mixto CSIC, Universidad de Sevilla, Americo Vespucio 49, Isla de la Cartuja, 41092 Seville, Spain; benitez@icmse.csic.es

**Keywords:** bio-waste, circular economy, cellulose nanofibers, chitosan, polylactic acid, mulching films

## Abstract

Biodegradable mulching films are a very attractive solution to agronomical practices intended to achieve more successful crop results. And, in this context, the employment of agricultural and industrial food residues as starting material for their production is an alternative with economic and environmental advantages. This work reports the preparation of bilayer films having two different wettability characteristics from three bio-derived biopolymers: TEMPO-oxidized cellulose nanofibers isolated from infused Yerba Mate residues, Chitosan and Polylactic acid. The infused Yerba Mate residues, the isolated and oxidized cellulose nanofibers, and the films were characterized. Nanofibrillation yield, optical transmittance, cationic demand, carboxyl content, intrinsic viscosity, degree of polymerization, specific surface area and length were studied for the (ligno)cellulose nanofibers. Textural and chemical analysis, thermal and mechanical properties studies, as well as water and light interactions were included in the characterization of the films. The bilayer films are promising materials to be used as mulching films.

## 1. Introduction

The materials used to create artifacts at a particular point in time were crucial in shaping and defining civilizations; thus, in analogy to other historical epochs like the Stone, Bronze, Iron, and Steel Ages, humanity’s current global culture can be defined by the term ‘Plastic Age’. In this context, materials are viewed as more than just physical substances; they are inseparable from the social and commercial systems they are deeply embedded in and, furthermore, they influence the ways in which people live, organize, and progress collectively. Therefore, understanding the materials characteristic of an era is essential for comprehending the larger social and economic dynamics of that period and the associated environmental impact.

Plastic’s mass consumption started less than a hundred years ago due to its ability to address global challenges in quite diverse fields, but, at the same time, it has given rise to new issues. The most widespread commercialized products are synthetic petrol-based polymeric materials, mainly as single-use plastics. The immense impact of plastic waste on ecosystems and human health highlights the need for a shift in material choices and consumption patterns. Emphasizing eco-friendly alternatives and promoting circular economies can help to mitigate the negative consequences of the Plastic Age while still acknowledging the significance of the materials shaping human civilization. In this sense, agricultural and industrial food residues play a crucial role in the circular economy by serving as valuable resources for extracting a variety of useful compounds, thus promoting sustainable practices and leading to economic and environmental benefits.

The dilemma of how to maintain the quality and style of life without continuing to damage the environment is not the only issue to solve. To satisfy the continuously growing worldwide demand, food production needs to be optimized while minimizing water consumption levels. Among the agronomic practices usually employed to achieve more successful culture results, mulching is commonly used. It has been applied using a variety of materials for over a thousand years [1]. In particular, the employment of plastic mulching films has made a revolution since they offer an evapotranspiration decrease and soil temperature control, thus leading to earlier crop maturation [2]. Additionally, pests are more easily controlled, and both the possible soil erosion and farming product losses due to rainfall are minimized [3]. Then, less herbicides and pesticides are used, a more efficient employment of water resources is achieved and, at the same time, humus formation increase is usually observed. As a result of this, mulching improves the soil’s organic and nutritional characteristics with a lower resource investment than in traditional culturing [1]. However, although plastic mulching has multiple benefits, some negative impacts must be considered. Since most plastic mulches are non-biodegradable and non-reusable, they should be removed from the fields, stockpiled, transported, or even burned. To analyze some statistics, each mulching hectare leads to approximately 72–259 kg of residual films [4]. Consequently, in addition to environmental damage, additional time and costs must be faced. Thus, developing and using biodegradable mulching films (BMF) appears to be a very attractive solution. Some biodegradable materials, such as Mater-Bi^®^ and Ecovio^®^, have been tested in tomato and melon crops, demonstrating the products’ superior yield and quality compared to those achieved by the employment of traditional mulching films [5].

Commonly, a single polymer is not able to meet all the required properties for a certain plastic product. Then, multilayer structures might offer an appropriate blend of characteristics and, in several cases, synergistic effects are observed [6]. Double- and triple-layer films containing poly-lactic acid (PLA) as a hydrophobizing agent, combined with hydrophilic components, have been reported as food packaging materials, whereas multilayer films mainly prepared from starch and latex were developed and studied for mulching purposes [7,8,9]. The obtained results indicated that the multilayer architectures are promising models in the development of new materials.

The extraction of biopolymers from residues brings about several positive ecological benefits as it prevents their accumulation as waste, decreases the reliance on non-renewable resources, and contributes to the reduction of non-biodegradable plastic waste. Furthermore, this practice is complementary to the food supply, not competing with it, and aligns perfectly with the principles of the circular economy. The primary green material that can be extracted from agricultural, forestry, and food residues is cellulose, the most abundant biopolymer on Earth. This biopolymer, along with its micro- and nanoscale forms, is biodegradable and lends itself well to the development of a wide range of materials [10,11,12,13,14,15].

A potential source of green residues for the extraction of cellulose includes not only those generated before but also after the consumption of Yerba Mate. The popular South American infusion is prepared by using hot water in a process that extracts a variety of bioactive substances from dry Yerba Mate leaves (produced by an evergreen subtropical tree). On average, an Argentinian citizen consumes up to 7.0 kg of Yerba Mate per year, whereas a Paraguayan citizen uses nearly 2.6 kg in the same period. In accordance to the Argentinian National Institute of Yerba Mate, Argentina is the main worldwide producer, having harvested more than 829 million kg of green leaves just during 2022, thus leading to over 275 million kg of packaged dry Yerba Mate ready to be consumed [16].

Some scientific research has been made about the use of the Yerba Mate harvest’s residues, as well as dry Yerba Mate leaves, to create high-added value products [17,18,19,20,21]. Some of the applications include the extraction of antioxidant substances, the production of energy, and their use as support for catalysts, among many others [22,23,24,25,26]. However, the material discarded from Mate infusions has only been considered by few researchers, though it represents millions of kilograms of a valuable starting material [22]. Very recently, a high-performance lithium–sulfur battery cathode was prepared from a carbonaceous material obtained after the pyrolysis of extracted cellulose from infused Yerba Mate residues [27]. As in the aforementioned example, it must be considered that cellulose and its derivatives are usually combined with other substances to overcome their fragile nature and their poor mechanical properties, as well as to successfully exploit their intrinsic properties.

On the other hand, due to its natural occurrence, abundance, availability and compatibility with cellulose, chitosan (CH) is often considered as a useful building block to produce engineered materials [28]. The second most abundant biopolymer, chitin is the predecessor to chitosan, which can be isolated from crustacean’s exoskeletons, usually discarded by fishing industries, and related ones. CH-based films tend to be very brittle, have poor absorption capabilities, and not usually be able to maintain their structure after being exposed to water. Then, the smart combination of CH with another biopolymer is highly desired. In this sense, nanocellulose (NC)/CH materials with enhanced properties can be achieved by leveraging the opposite charge of the starting materials to create polyelectrolyte complexes (PECs) [29]. Additionally, since both NC and CH are β-glucans, these polymers also have the capability to form stable structures through specific hydrogen bonds facilitated by their structural and entropic behavior in water solutions [30,31,32,33]. These unique interactions obviate the need for additional substances, like crosslinkers.

In the present research work, and to the best of our knowledge, for the first time, TEMPO-oxidized cellulose nanofibers (TO-YCNF) were isolated and prepared from infused Yerba Mate residues. The TO-YCNF nanofibers were exhaustively characterized and integrated with CH to improve the mechanical and water-absorption properties of the hydrophilic side of an environmentally friendly bilayer film. CH will provide some water uptake capacity and fungi proliferation control during the in-service performance (a common BMF issue) [34]. Additionally, CH is expected to act as a natural elicitor and a later source of nitrogen for soil during the film biodegradation step (it has been observed that the presence of residual plastic films in agricultural soils could lead to a reduction in inorganic nitrogen contents and functional enzyme activity in soil, and affect the nitrogen cycling) [35,36,37]. On the other hand, and principally to satisfy the water permeability requirements, the hydrophobic layer was prepared from PLA.

Such bilayer films are expected to have the required properties to replace conventional mulching plastics.

## 2. Materials and Methods

### 2.1. Materials

Infused Yerba Mate residues were produced from commercial brands. The reagents used for cellulose nanofiber production were Sodium Hydroxide (NaOH, ≥97%), Sodium Chlorite (NaClO_2_, >80%), Acetone, Acetic Acid (ACS reagent, ≥99.7%), (2,2,6,6-Tetramethyl-1-piperidinyl) oxidanyl (TEMPO, free radical, 98%) from Sigma Aldrich, Sodium Bromide (NaBr, 99%) and Sodium Hypochlorite (NaClO, 80%) supplied from Honeywell, Fluka^TM^ (Morris Plains, NJ, USA). Furthermore, the reagents used for films preparation and characterization were Chitosan (Low molecular weight, Sigma-Aldrich, St. Louis, MO, USA), Polylactic acid (6060D, NatureWorks, Minneapolis, MN, USA), Acetic Acid (PanReac, ≥99.7%, ITW Reagents, Barcelona, Spain) and Ethyl Acetate (Honeywell Riedel-de Häen, ≥99.7%).

### 2.2. Methods

**Scheme 1:**
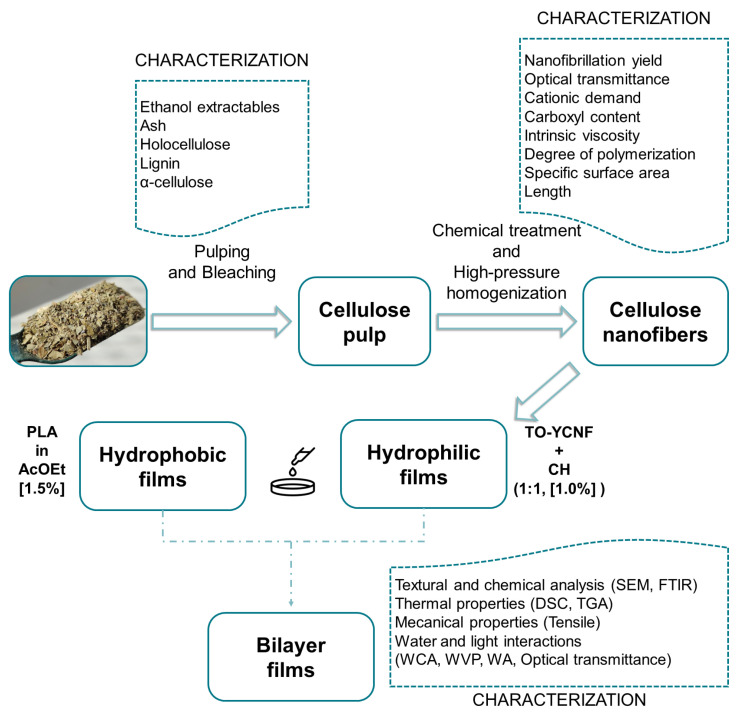
Workflow for the preparation and study of the bilayer films.

Scheme 1 shows the workflow followed along the present research work. Briefly, cellulose nanofibers were isolated from infused Yerba Mate residues and then they were used for the formulation of bilayer films together with chitosan and polylactic acid. The detailed methodology followed, as well as the characterization tests performed, are described in this section. All characterizations were made in triplicate, unless specified.

#### 2.2.1. Nanocellulose Preparation and Characterization

Cellulose nanofibers were isolated from dried and previously infused Yerba Mate residues, which were subjected to an alkaline process that allows high processing yields to be obtained, in addition to being environmentally friendly by using a 15 L rotary reactor (Metrotec S.A., Cordoba, Spain) under the following conditions: 7% NaOH on dry raw material, a liquid/solid ratio of 10/1 at 100 °C during 150 min. Once chemically treated, the mixture was passed through a disintegrator (Metrotec S.A.). To achieve the fiber fibrillation, the material was processed in a Sprout-Bauer defibrillator (Combustion Engineering, Springfield, OH, USA), whereas the resulting suspension was sieved to obtain cellulose pulp using a 0.14 mm mesh [38].

Three subsequent bleaching treatments were applied on the cellulose pulp (50 g of dry pulp in 1600 mL of water) by adding 15 g of sodium chlorite and 5 mL of concentrated acetic acid to generate chlorine dioxide at 75–80 °C in a water bath.

The chemical characterization of the raw materials on their ethanol extractables, lignin, holocellulose, ash and α-cellulose, was done according to TAPPI standards T-204 cm-97, T-222 om-02, T9m-40, T-211 om-02 and T-429 cm-10, respectively [39,40,41,42,43].

TO-YCNF was obtained from bleached cellulose pulp using an oxidation pretreatment with 5 mmol/g of NaClO at 10% as oxidizing agent and TEMPO catalyst while maintaining a medium pH = 10.2. Once the addition of the oxidant was finished, the pH was kept constant by the addition of 0.5 M NaOH, and the reaction was allowed to run for 2 h. The process was stopped by the addition of 100 mL of ethanol, and the resultant product was then washed with distilled water [44]. The suspension obtained (concentration of approximately 1%) was subjected to a high-pressure homogenization process of 4 cycles at 300 bar, 3 cycles at 600 bar and 3 cycles at 900 bar pressure in a Panda GEA 2K Niro equipment (GEA Group, Düsseldorf, Germany) [38].

The nanofibrillation yield of TO-YCNF was gravimetrically determined by centrifuging a 0.1% suspension at 10,000 rpm for 20 min. The sediment fraction was recovered, weighed and oven-dried at 90 °C until constant weight [38]. The optical transmittance at TO-YCNF 0.1% suspension was measured from 400 to 800 nm (T 800) on a Perkin Elmer Lambda 25 UV spectrometer (Shelton, CT, USA). Cationic demand (CD) was determined using a Particle Charge Detector (Mütek PCD 05, Montreal, QC, Canada) and following the well-known methodology described in the literature [45]. Regarding the carboxyl content (CC), it was measured by conductimetric titration in accordance with the methodology proposed by Besbes et al. [44]. The intrinsic viscosity ($\eta_{s}$), the value that is related to the degree of polymerization (DP, Equation (1)) of the TO-YCNF, was determined according to ISO 5351:2010 [46]:

(1) $$DP\left(<950\right):DP=\frac{\eta_{s}}{0.42}$$

Then, the length of the nanofibers could be calculated from the DP using the equation described by Shinoda et al. (Equation (2)) [47]:

(2) $$Length\left(nm\right)=4.286\cdot DP-757$$

The theoretical specific surface area ($\sigma_{CNF})$ was obtained from the Equation (3) and the known surface area of the polydiallyldimethyl ammonium chloride (Poly-DADMAC), the cation used in the determination of the CD [48]. For this purpose, a monolayer interaction of the poly-DADMAC on the fiber’s surface was assumed.

(3) $$\sigma_{CNF}=\left(CD-CC\right)\cdot \sigma_{\mathrm{Poly-DADMAC}}$$

where $\sigma_{\mathrm{Poly-DADMAC}}$ corresponds to the surface area of a single poly-DADMAC molecule (4.87·1017 nm^2^/µeq). Then, assuming that the TO-YCNF are cylindrical, their diameter (d) was calculated using Equation (4), where the fiber density was assumed to be 1600 kg/m^3^ [48,49,50].

(4) $$d=\frac{4}{\sigma_{CNF}\cdot 1600\cdot \frac{kg}{m^{3}}}$$

#### 2.2.2. Bilayer Films Preparation and Characterization

The hydrophobic layer was first prepared from a PLA solution (1.5 wt% in Ethyl Acetate) by being cast (20 mL) in a Petri dish. Once the PLA film was air-dried, the hydrophilic layer was built onto it by pouring the CH/TO-YCNF mixture prepared based on the experience of our previous studies [51]. For this, equal mass quantities (15 g) of a CH solution (1.0 wt% in acetic acid 1 *v*/*v*%) and the TO-YCNF suspension were mixed during 3 min at 10,000 RPM in an Ultra-Turrax homogenizer (IKA T18 digital Ultra Turrax, Wilmington, NC, USA). The hydrophilic layer was allowed to dry in room conditions for 48 h. As references, single blank hydrophobic, hydrophilic, TO-YCNF and CH films were prepared using the corresponding solutions/suspensions and following an analogous procedure. The film’s thickness was measured using a Digital Micrometer IP65 0-1″ (Digimatic, Mitutoyo, Tokyo, Japan).

The material morphology was studied by scanning electron microscopy (SEM) in a Jeol JSM6490LV instrument, (Tokyo, Japan). All the samples had been previously sputtered with a thin gold layer. To study their internal morphology, some of them were cryo-fractured after their immersion in liquid N_2_.

The chemical characterization was done by Attenuated Total Reflectance Infrared Spectroscopy (ATR-IR) with a diamond crystal single reflection accessory (MIRacle, Pike, Memphis, TN, USA) coupled to an FTIR spectrometer (4100 FT-IR, Jasco, Oklahoma City, OK, USA), 128 scans in the 700–4000 cm^−1^ range at 4 cm^−1^ resolution were accumulated. The spectra obtained were baseline-corrected and normalized.

The thermal behavior of samples was analyzed by Differential Scanning Calorimetry (DSC) (Q20, TA Instruments, New Castle, DE, USA) using non-hermetic aluminum pans (TZero, TA) in a heat/cool/heat cycle from −70 to 175 °C under N_2_ flow (50 mL/min), and by Thermogravimetric Analysis (TGA) (METTLER TOLEDO, Columbus, OH, USA) from 30 to 750 °C heating at 10 °C/min rate and under a N_2_ flow of 50 mL/min.

Tensile tests were carried out with a universal testing machine (Criterion 42, MTS, Eden Prairie, MN, USA) at a deformation speed of 5 mm/min. Samples were dumbbell-cut with a crosshead distance of 20 mm. Tensile parameters were referred to the initial sample cross-section and length.

Static Water Contact Angle (WCA) values were determined with an optical tensiometer (Attension TL100, KSV, Helsinki, Finland) in sessile drop mode and using 2 µL Milli-Q grade water drops. The drop contour was monitored for 20 s and analyzed using the OneAttension (Biolin Scientific, Gothenburg, Sweden) built-in software.

The water vapor absorption (WVA) of each sample was also determined. For this, dry samples were weighed before and after being placed under different RH conditions for 24 h (≤10, 30, 60, 80 and ≥90%). The amount of adsorbed water vapor was calculated taking the initial dry weight as the reference (Equation (5)):

(5) $$WVA\left(\%\right)=\frac{W_{f}-W_{i}}{W_{i}}\times 100$$

where W_i_ and W_f_ are the weights of the dried films before and after being placed under the different RH conditions, respectively [4].

The water vapor permeability was determined at 25 °C and under a 100% relative humidity gradient. In this test, permeation chambers with an interior diameter of 0.70 cm and 1.30 cm of inner depth were used and filled with 350 μL of deionized water. The samples were cut into circles and mounted on the top of the permeation chambers, which were further placed in a desiccator. The water vapor transferred through the film was determined from the mass loss of each permeation chamber every hour during a period of 7 h and after stabilization. Then, the obtained results were plotted as a function of time, and the slope referred to the permeation area to determine the water vapor transmission rate (WVTR) (Equation (6)):

(6) $$WVTR\left(\frac{g}{m^{2}\cdot day}\right)=\frac{W_{CHt}-W_{CHi}}{\Delta t\cdot A}$$

where W_CHt_ and W_CHi_ are the weights of the chambers at t and zero time, respectively, t refers to the time and A corresponds to the film’s area [52].

The optical properties of the films were studied by recording the transmittance spectra in the 200–800 nm range using a UV-Vis spectrometer (Cary 300, Agilent, Santa Clara, CA, USA) equipped with an integrating sphere (LabSphere, North Sutton, NH, USA).

## 3. Results

### 3.1. Nanocellulose Fibers Isolation and Characterization

The infused Yerba Mate residues and the bleached and unbleached products after the pulping process were characterized (Table 1). The inorganic (ashes), extractables and hemicellulose contents were progressively reduced from the raw material (3.76%, 27.33% and 29.23%, respectively) to the unbleached (2.45%, 21.26% and 25.35%, respectively) and bleached pulps (1.89%, 17.23% and 20.12%, respectively). On the other hand, the α-cellulose content increased from 35.63% in the infused Yerba Mate residues to 43.53 and 57.54% in the unbleached and bleached pulps, respectively. This can be explained considering that the pulping process reduces the total mass of residues but leaves most of the cellulose intact. A similar concentration effect took place with lignin considering the raw material and the unbleached pulp but, as can be seen, in the bleached pulp, the lignin content was successfully reduced.

Then, TO-YCNF was prepared from the bleached pulp, and it was fully characterized (Table 2). The nanofibrillation yield (61%) was similar to others obtained when a mixture of vegetable residues was employed as starting raw material for TO-CNF, whereas the optical transmittance (52%) was smaller than in the mentioned study [51]. The transmittance of the CNF suspension (T_800_) is directly related to the nanofibrillation degree: as the nanofibrillation yield decreases, the optical transmittance diminishes due to the poor light dissipation achieved by the fibers [38]. The interaction of the anions on the fibers’ surface with the external nearest area is high since the CD value is 1492 µeq/g, indicating the effectiveness of the TEMPO-mediated oxidation treatment for the introduction of carboxyl groups. The obtained value is higher than the one observed for similar TO-CNF obtained from different vegetable residues [51,53,54]. The specific surface of the TO-YCNF (634 m^2^/g) is higher than the one reported for other TO-CNF, whilst the CC (190 µeq/g) value is intermediate, thus revealing the success of the TEMPO-mediated oxidation treatment [51,53,54]. Regarding the fibers’ morphology, their length (798 nm) is between other values reported for TO-CNF prepared by the same procedure from a mixture of vegetable residues (614 nm) and wheat straw (1033 nm), whereas their diameter is smaller (3.94 nm) than the one observed in the mentioned cases (5–6.81 nm) [51,53].

### 3.2. Bilayer Films

**Figure 1:**
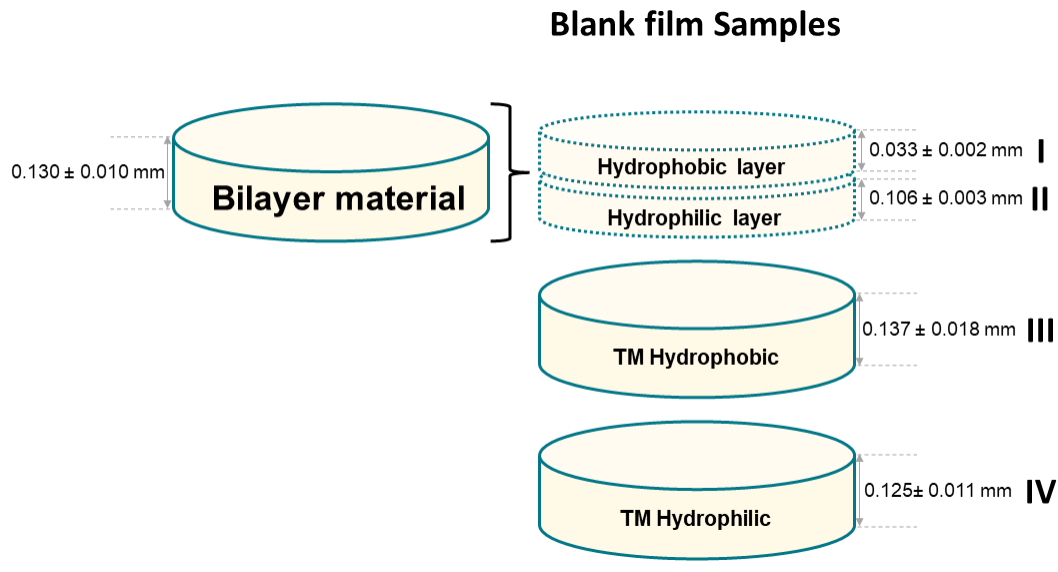
Thickness values of all prepared films.

The thickness of the prepared bilayer film was designed considering the thickness of other mulching materials. Unless for solarization purposes, this aspect does not affect the mulching result. Additionally, four different blank samples were prepared to be compared with the bilayer film (Figure 1): hydrophobic (I) and hydrophilic (II) monolayer films whose thicknesses were comparable to the individual ones present in the bilayer material (≈0.033 and 0.106 mm, respectively), and other hydrophobic (III) and hydrophilic monolayer (IV) films whose thicknesses have similar values to the total thickness of the bilayer film (TM samples, ≈0.130 mm).

#### 3.2.1. Textural and Chemical Analysis

**Figure 2:**
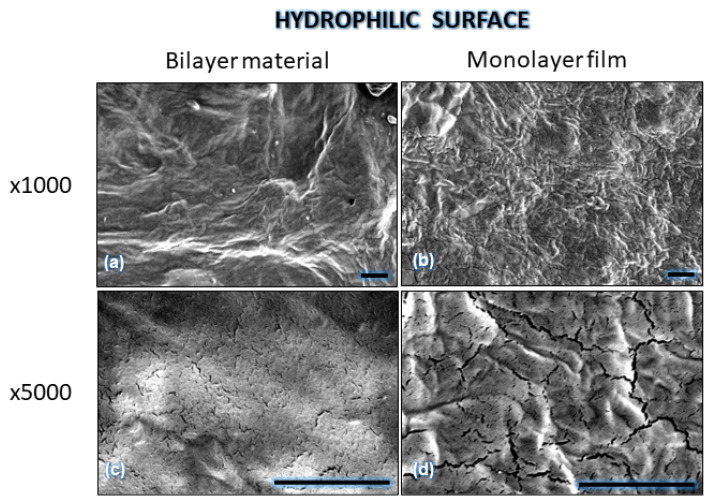
SEM micrographs of the films’ hydrophilic surface: bilayer film ((**a**) ×1000 and (**c**) ×5000) and monolayer hydrophilic film ((**b**) ×1000 and (**d**) ×5000). Scale: 10 µm.

**Figure 3:**
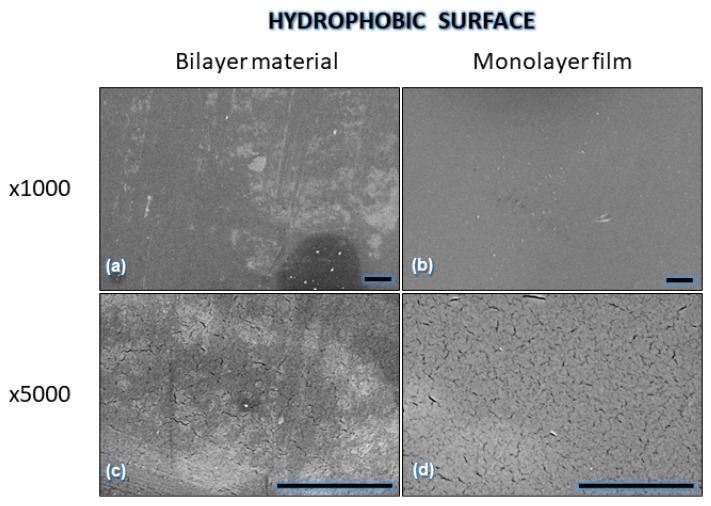
SEM micrographs of the films’ hydrophobic surface: bilayer film ((**a**) ×1000 and (**c**) ×5000) and monolayer hydrophobic film ((**b**) ×1000 and (**d**) ×5000). Scale: 10 µm.

**Figure 4:**
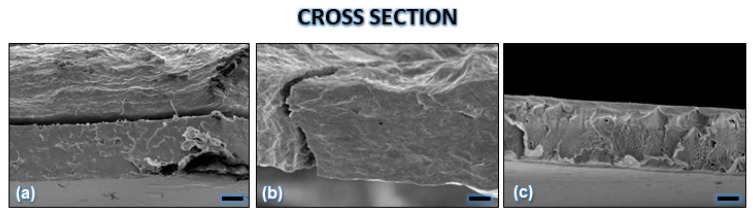
SEM micrographs of the films’ cross-section: (**a**) bilayer film, (**b**) monolayer hydrophilic film and (**c**) monolayer hydrophobic film. Scale: 10 µm.

SEM micrographs of the prepared materials were analyzed to better understand the morphology of films (Figure 2, Figure 3 and Figure 4). Both surfaces of the bilayer material were irregular, but the hydrophilic one (Figure 2) showed a more noticeable roughness than the hydrophobic one (Figure 3). These characteristics are frequent in polysaccharides-based mulching materials [34,55]. The micrographs of the monolayer materials showed the same general features. In these cases, the hydrophobic surface texture is very similar to the one present in the bilayer film, whereas the hydrophilic one is rougher. From the cross-sectional images, it is possible to observe that, in several cases, cracks present in the monolayer hydrophilic material were dividing the films across their entire thickness (Figure 4). Oppositely, the cracks present in the monolayer hydrophobic material were just superficial. Regarding the bilayer film, the cracks’ sizes were significantly reduced and seemed to be superficial. Cross-section images also revealed an interface separation between the hydrophilic and hydrophobic sides of the bilayer film. At this point, authors cannot elucidate whether this behavior is due to the cryo-fracture processing or not.

**Figure 5:**
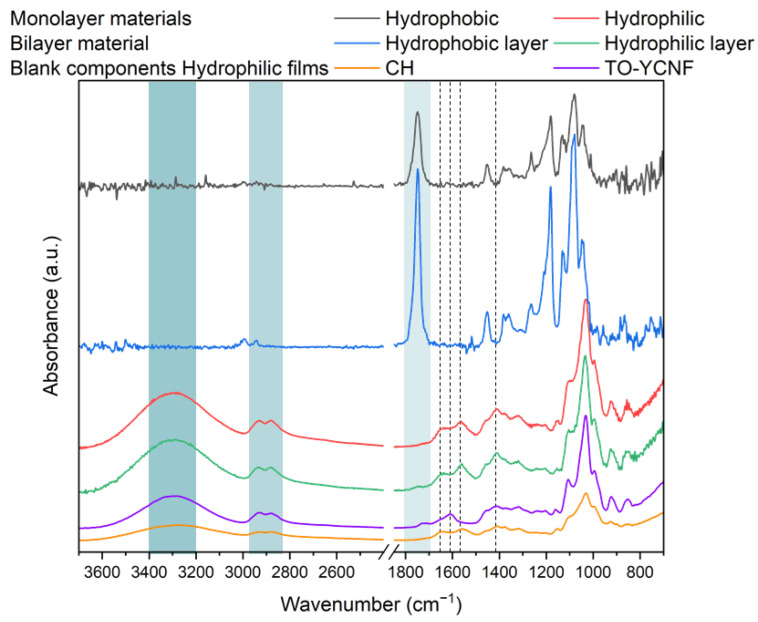
FTIR spectra of films prepared from just the starting materials (TO-YCNF and CH), and of the prepared mono- and bilayer films. The bilayer film was irradiated by the hydrophobic and hydrophilic sides, respectively.

The chemical characterization of films, either as monolayers or in the bilayer format, has been carried out by ATR-FTIR (Figure 5). Those corresponding to the pristine CH matrix and to the TO-YCNF nanofibers are included as references. Bands for TO-YCNF were observed at 3200–3400 cm^−1^ due to the -OH groups, near to 2900 cm^−1^ corresponding to -CH stretching and at 1600 cm^−1^ being related with the presence of -C=O functional groups as a result of the TEMPO oxidation treatment [54]. On the other hand, the CH showed a broad band centered at 3300 cm^−1^ attributed to both -OH and -NH groups, another one near to 2900 cm^−1^ because of the -CH groups, whereas the ones at 1659 cm^−1^ and 1555 cm^−1^ corresponded to the Amide I and Amide II bands, respectively [28]. The hydrophilic monolayer displayed a mixture of bands from CH and TO-YCNF and no features suggesting the formation of new covalent linkages. However, there is evidence about ionic interactions in the PECs formed: the absence of the TO-YCNF band at 1600 cm^−1^ and the appearance of the 1638 cm^−1^ band indicate an electrostatic interaction between CH and TO-YCNF. These results are similar to the ones observed by other authors in similar PEC materials [56]. Otherwise, the hydrophobic monolayer presented the characteristic bands of PLA, including the 1750 cm^−1^ and 1180 cm^−1^ ones related to both -C=O and C-O-C stretching [57].

The bilayer material was studied analyzing both the hydrophilic (CH/TO-YCNF) and hydrophobic (PLA) sides and no differences with the corresponding monolayer films were observed. Such a result indicates that there is no chemical interaction between both layers. Similar behavior was observed in other casting-prepared bilayer films containing PLA and pea starch [8].

#### 3.2.2. Mechanical and Thermal Characterization of Films

**Figure 6:**
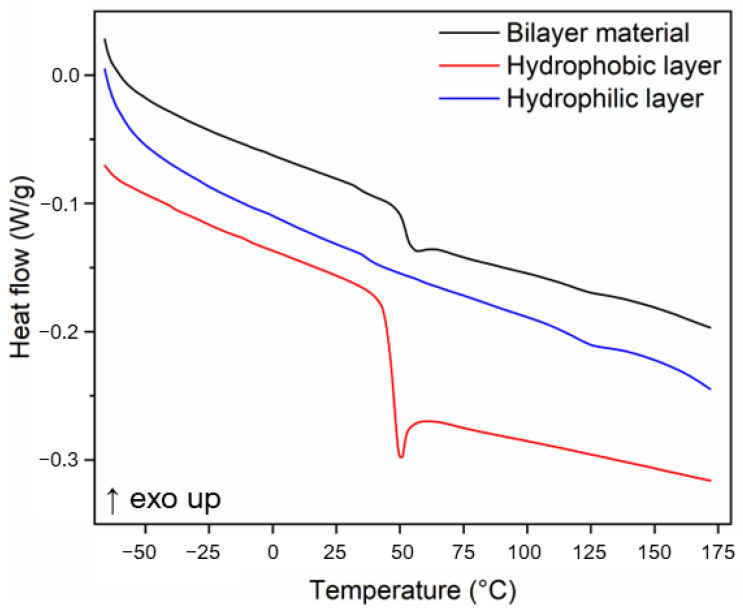
DSC thermograms of the mono- and bilayer films.

The thermal behavior and stability of films were studied by DSC and TGA, respectively. As expected, the hydrophilic (CH/TO-YCNF) showed no intrinsic melting event as the melting temperature of its components (chitosan and TEMPO-oxidized cellulose nanofibers) is above 175 °C and beyond their thermal decomposition (Figure 6). The weak endothermic peak around 125 °C is very likely due to some solvent residue after their preparation by casting. On the other hand, the hydrophobic layer lacks the characteristic exothermic crystallization around 127 °C and the melting at 157 °C observed in semi-crystalline PLAs [58]. This result indicates that the hydrophobic layer is amorphous. The thermogram of the bilayer was essentially the sum of those of the individual monolayers, which suggests the lack of chemical/structural interaction between the monolayers. Interestingly, the glass transition temperature of PLA in the bilayer film is about 5 °C higher than in the monolayer (Table 3). This can be due to the migration of a small amount of some component of the hydrophilic monolayer acting as a filler in the hydrophobic one.

**Figure 7:**
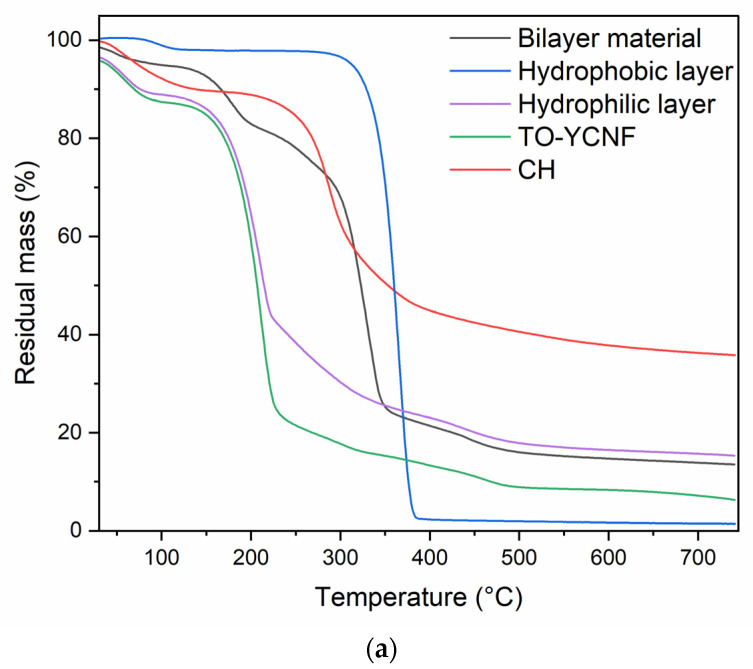

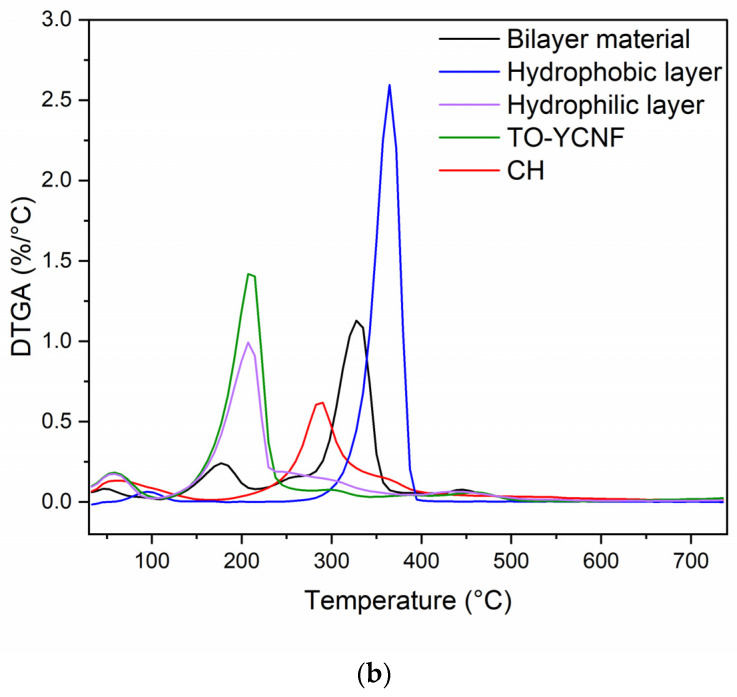
(**a**) TGA thermograms of the mono- and bilayer films and (**b**) their corresponding first derivatives (DTGA).

The degradation behavior of the films during the heating processes is shown in Figure 7. From room temperature to 150 °C, all samples lost free and bonded water molecules, and some of them might also have lost some solvent residues (acetic acid), whereas degradation events took place from 120 to 500 °C. The temperature at which each degradation rate is fastest, T_max_, is clearly observed in the DTGA curves, (Figure 7b) being 327, 364, 207, 207 and 287 °C for the bilayer material, the hydrophobic layer, the hydrophilic layer, TO-YCNF and CH films, respectively. CH and the hydrophobic layer both presented thermal events in accordance with those previously observed [51,59,60,61]. On the other hand, TO-YCNF and the hydrophilic layer presented the same T_max_ value, which is smaller than others reported for TO-CNF prepared from cotton stalks (near 230 °C) and from a vegetable residue mixture (two main events with similar intensities at 234 and 291 °C) [51,62]. This phenomenon is mainly related to the CNF’s diameters: a lower fiber diameter is associated with a higher specific surface that can be exposed to heat, thus promoting an earlier thermal degradation [54]. The degradation temperature associated with chitosan’s degradation in the hydrophilic sample is lower than the T_max_ value observed for the blank CH film, which indicates interaction between the CH and the TO-YCNF. The bilayer material presented two main thermal events (177 and 327 °C) directly associated with the individual layers constituting it, and in both cases, the maximums are located at lower temperatures than in the individual layers. Similar thermal properties were observed for a bilayer film composed of isolated soy protein and PLA, and its corresponding monolayer films [61].

**Figure 8:**
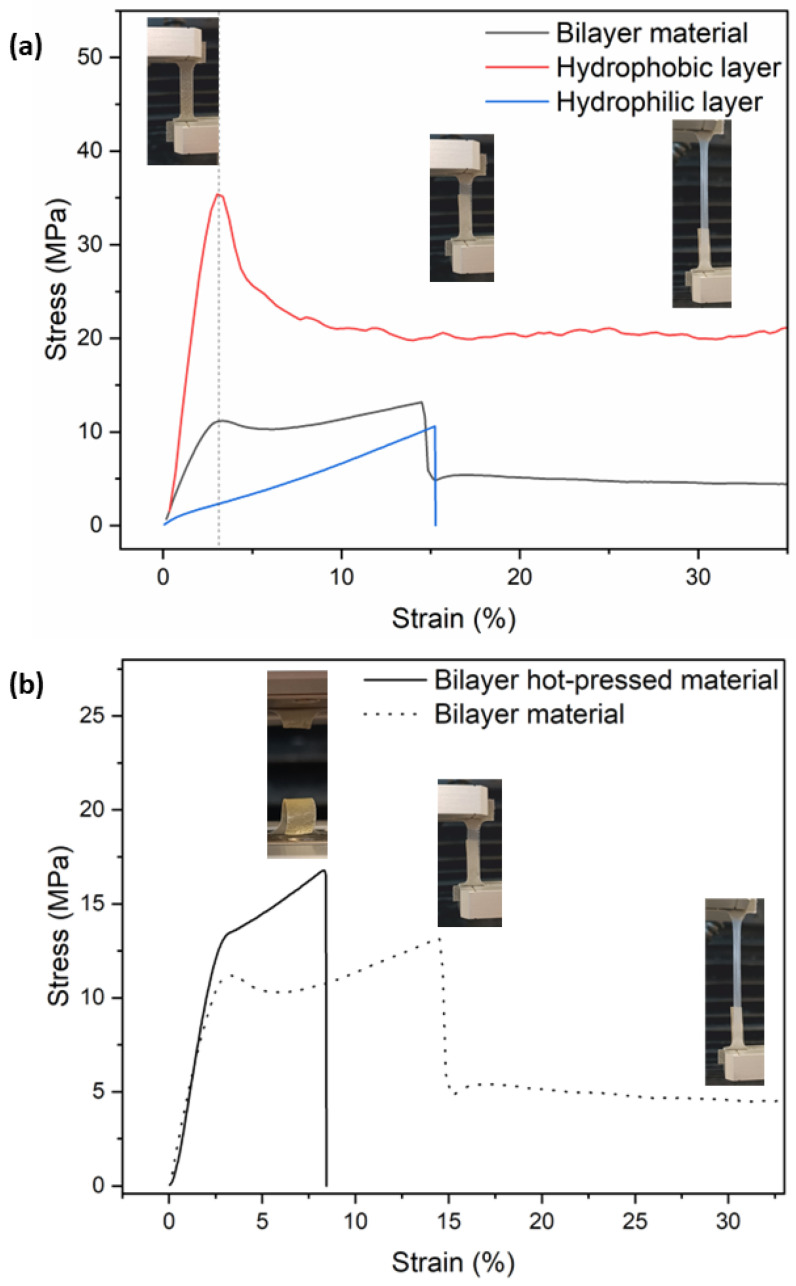
(**a**) Tensile tests of the mono- and bilayer films; (**b**) Tensile tests of both the original and the hot-pressed bilayer material.

Tensile parameters for the complete series of samples prepared are summarized in Table 4. The mechanical behavior of the two monolayer components of the bilayer film is quite different (Figure 8a). While the hydrophilic monolayer (CH/TO-YCNF) exhibited an elastic response with a Young’s modulus of about 100 MPa and a breaking strain of ~17%, the hydrophobic monolayer (PLA) was much more rigid (Young´s modulus ~1700 MPa) and showed a plastic pattern with a yield point at 3.8%, followed by necking and a cold-drawing stage before rupture around 65% elongation. The behavior of the bilayer film was due to the combination of its hydrophobic and hydrophilic components, which suggests poor interaction between them. Indeed, the braking of the hydrophilic side of the bilayer was observed at the same elongation as the isolated hydrophilic monolayer, while the hydrophobic side continued its deformation following the pattern of the hydrophobic monolayer. The lack of interaction between both sides could also be deduced from the values of Young’s modulus. The one for the bilayer (Eb) can be theoretically calculated from those of the individual layers (E_1_ and E_2_) and their thicknesses (L_1_ and L_2_) according to Equation (7):

(7) $$E_{b}=\frac{E_{1}\cdot L_{1}+E_{2}\cdot L_{2}}{L_{1}+L_{2}}$$

With parameter values in Figure 1 and Table 4, the predicted modulus for the bilayer should be 483 MPa, which corresponds with the experimental one of 578 + 125 MPa.

The lack of adherence and interaction between both sides of the bilayer film was already suggested by the observed gap in cross-sectional SEM images and by ATR-FTIR results. Then, to improve the layer’s adhesion, some bilayer films were hot-pressed (70 °C, 4 tons, 10 min) and further evaluated (Figure 8b). As a result of this extra treatment, it was observed that, although the material did not delaminate, the mechanical parameters did not improve or change substantially, and no new chemical interactions were observed by FTIR.

#### 3.2.3. Film Interaction with Water and Light

The hydrodynamic properties, as well as the light transmission, are key parameters for films intended for mulching. One of the main utilities of using mulching films is related to their capability to boost water efficiency by preventing the evapotranspiration processes. Additionally, reducing weed growth and minimizing soil compaction are also important aims. Moisture loss is diminished when the film acts as a protective top layer for the soil. Furthermore, mulching films should be impermeable to allow water to flow off, resulting in nutrient retention near the roots while minimizing the leaching of fertilizers/herbicides/agrochemicals. In addition to this, the obstruction of UV light and photosynthetically active radiation are also highly desirable for the prevention of weed growth [4].

**Figure 9:**
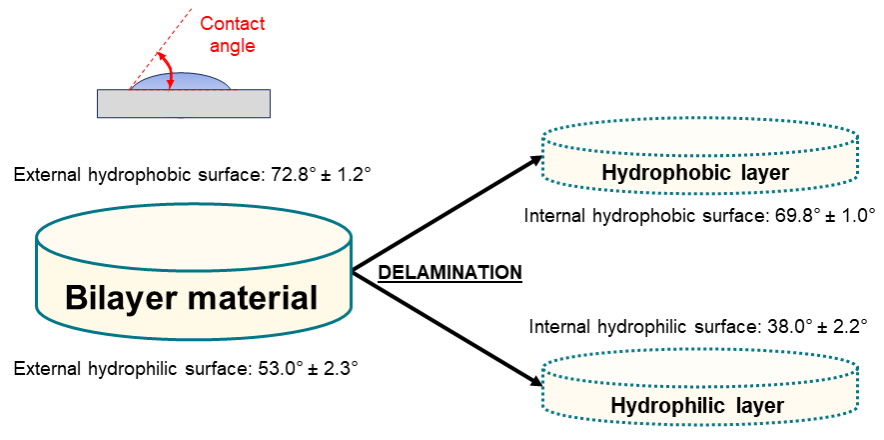
WCA of the external and internal surfaces of the bilayer material.

Film surface hydrophobicity/hydrophilicity has been evaluated by WCA measurements (Figure 9). Layers can be considered hydrophilic, as all WCA values are below 90°. The PLA side of the bilayer film has a WCA of 73 + 1°, close to the 75–85° reported range [63]. However, the CH/TO-YCNF side is much more hydrophilic (WCA 53 + 2°). Chitosan WCA have been reported to decrease with contact time and values after 20 s are around 75° [64]. It is evident, then, that the addition of TO-YCNF has provided hydrophilicity to the layer surface. In an additional experiment, the WCA of contacting internal surfaces after bilayer delamination under mechanical stress has been measured. In this case, the value for the PLA side is 70 + 1° while the one for the CH/TO-YCNF side has decreased up to 38 + 2°. Such results suggest that some residual water from the casting solution remains between the hydrophobic and hydrophilic layers of the bilayer film, and it is very likely the cause of the poor adherence between them.

**Figure 10:**
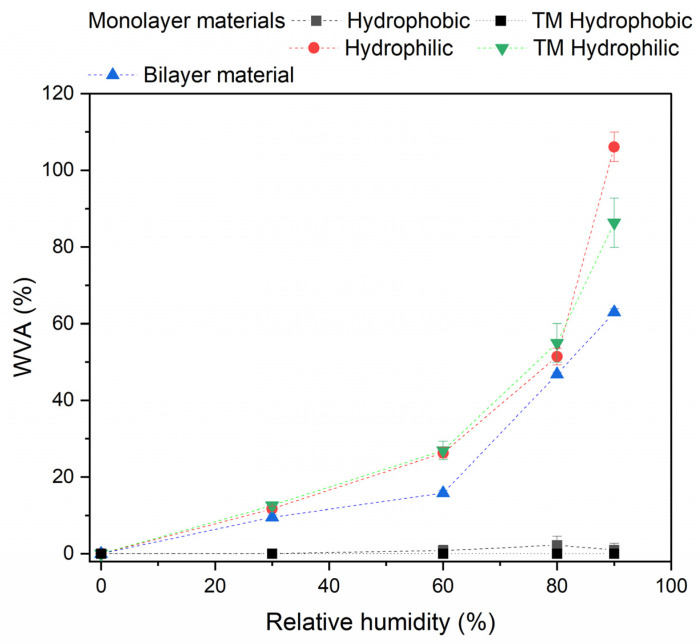
Water vapor absorption tests of the prepared mono- and bilayer films.

The water absorption capacity of samples under different RH conditions has also been tested (Figure 10). As was expected, the TM and the monolayer hydrophobic films are almost unable to uptake water vapor at any environmental condition. On the other hand, the monolayer and TM hydrophilic films retain high water vapor quantities. The bilayer film behavior was intermediate but closer to the hydrophilic profile because the size of the hydrophobic component is much smaller than the hydrophilic one. In any case, the water absorption capacity peaks up to ~60% at 90% RH. Considering that the material’s moisture content is an important factor influencing the biodegradation process, since it can promote the enzymatic degradation as well as serve as a humidity buffer for the plant, once again, the obtained results are promising for the intended application [65].

The water vapor permeability values of films are compiled in Table 5. Results showed the enormous difference of incorporating the hydrophobic layer in the bilayer film. Even though the thick (TM) and thin monolayer hydrophilic films are relatively permeable, this property is highly reduced with the incorporation of a thin PLA layer (0.033 mm). The permeability of the bilayer film (6.9 × 10^−6^ g/m d Pa) is consistent with the theoretical value (8.2 × 10^−6^ g/m d Pa) calculated from the contribution of the two layers according to Equation (8)

(8) $$P_{T}=\frac{L_{T}}{\frac{L_{1}}{P_{1}}+\frac{L_{2}}{P_{2}}}$$

where P_T_ and L_T_ are the permeability and thickness of the bilayer film, respectively, while L_i_ and P_i_ are those of the stacked layers. Values are comparable to those of commercial bio-based BMF such as Mater-Bi^®^, Ecovio^®^ and Ecoflex^®^, as well as other PLA/PBAT composites (1–4 × 10^−6^ g/m d Pa) [66,67]. These results are promising if considering the looked-for effect of mulching films on the retention of the soil’s water.

**Figure 11:**
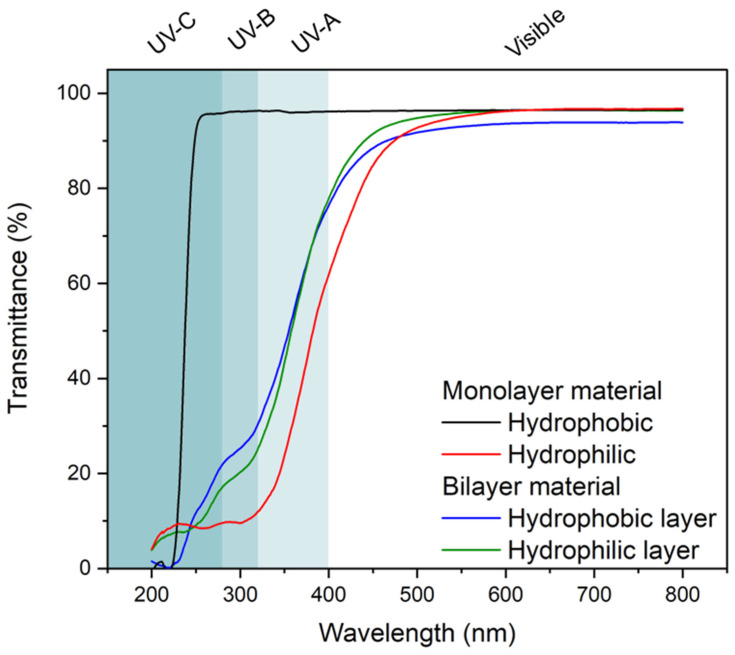
UV–Visible transmittance of the prepared mono- and bilayer films. The bilayer film was irradiated by the hydrophobic and hydrophilic sides, respectively. UV-C, UV-B, UV-A, and Visible regions of the electromagnetic spectrum are indicated by using shades of blue and white colors in the figure.

Finally, the transmittance of the films in the ultraviolet and visible regions was evaluated (Figure 11). In general, bilayer and monolayer films had a low UV–Vis blocking capacity, particularly the hydrophobic monolayer film. On the other hand, the hydrophilic layer was able to block most of the UV-C and UV-B incident light (Transmittance ≈ 10%) while keeping a moderately high shielding of UV-A. This difference might be attributed to (a) the presence of residual aromatic compounds in the hydrophilic layer, such as lignin (able to absorb light at values near to 280 nm) [68,69,70], and (b) to the higher surface roughness that generates diffuse light scattering [71]. The performances of the bilayer film are the combination of both components and benefits from the contribution of the hydrophilic layer. Thus, the shielding capacity in the UV region is highly improved with respect to the single PLA component.

The UV–Vis transmittance of the bilayer film is slightly different depending on the side from which it is irradiated. Thus, visible light transmittance is lower if irradiated from the hydrophobic layer because of the specular reflection caused by the smoother surface texture. In the UV region, such behavior is inverted, and shielding is better if irradiated from the hydrophilic side. A possible explanation for this is the occurrence of fluorescence caused by residual aromatic compounds and a better scattering from outermost layers. This property can be significantly useful for protecting photo-sensitive soil additives in case they are needed.

These results are promising when considering the film’s possible mulching use because visible light is necessary for the photosynthesis while protection from harmful UV is required.

## 4. Conclusions

TO-YCNF has been successfully prepared from infused Yerba Mate residues. The nanofiber diameters were very small (3.94 nm), thus determining a particular set of characteristics. Their combination with CH has led to a hydrophilic layer, which, when combined with the relatively hydrophobic layer prepared from PLA, resulted in the formation of bilayer films. The bilayer film’s properties showed some synergistic effect as a result of the material’s combination, making it suitable for mulching use. The bilayer film’s permeability was 6.9 × 10^−6^ g m/m^2^ d Pa, whereas the optical transmittance from UV to 400 nm was lower than 75%. The WVA absorption was higher than 60% at 90% of relative humidity, the elongation at break was 13.9% and the material suffered delamination. The SEM and FTIR studies revealed that the individual layers were not chemically interacting with each other. The WCA-obtained values showed that the two sides of the bilayer films have different wettability properties, as was expected.

The bilayer film’s characteristics are promising regarding its possible use as mulching material. Soil biodegradation characteristics and the effect of their weather exposure are currently being studied. In future studies, and to be more environmentally friendly, the hydrophobic layer would be prepared without the use of the organic solvent considered here.

## Figures and Tables

**Table 1:** Chemical characterization of the raw material and the bleached pulp.

Component	Infused Yerba Mate Residues (%)	Unbleached Pulp (%)	Bleached Pulp (%)
Ashes	3.76 ± 0.13	2.45 ± 0.21	1.89 ± 0.19
Extractables	27.33 ± 1.34	21.26 ± 1.11	17.23 ± 1.63
Lignin	4.05 ± 0.41	7.41 ± 0.45	3.22 ± 0.3
Hemicellulose	29.23 ± 3.31	25.35 ± 3.41	20.12 ± 2.21
α-Cellulose	35.63 ± 3.25	43.53 ± 4.92	57.54 ± 4.39

**Table 2:** TO-YCNF main characterization results, and bibliographic comparative.

Sample	Yield (%)	T_800_ * (%)	CD (µeq/g)	CC (µeq/g)	Length (nm)	σ_specific_ (m^2^/g)	Diameter (nm)	DP
TO-YCNF	61.67 ± 3.5	52.3	1492.4 ± 41.43	190.41 ± 18.76	798	634	3.94	362.81
TO-CNF [51]	63.44 ± 4.52	74.7	1043.54 ± 18.2	148.12 ± 5.26	614.26	436	6	319.94
TO-CNF [53]	96.4 ± 0.4		1440.1 ± 20.1	369.5 ± 2.9	1033	521	5	
TO-CNF [54]	98.7	90	1116.5 ± 43.1	367.0 ± 8.72		367.01	6.81	502

* TO-YCNF suspension concentration: 0.9608% *w*/*w*.

**Table 3:** Thermal parameters extracted from DSC analysis.

Sample	1st Heating		Cooling	2nd Heating	
	T_g_ (°C)	Endothermic Peak	Tg (°C)	Tg (°C)	Endothermic Peak
Bilayer	39.6	-	47.5	53.2	125.5 °C * 0.444 J/g
Hydrophobic monolayer	35.6	-	42.8	47.9	-
Hydrophillic monolayer	-	-	-	36.8	125.2 °C * 0.508 J/g
TM Hydrophobic	24.1 *, (61.2)	93.9 °C * 2.48 J/g	41.6	45.6	-
TM Hydrophilic	-	-	-	35.1	125.6 °C * 0.683 J/g

Parameters in parentheses indicate minor events. (*) Thermal events associated with preparation residues.

**Table 4:** Average tensile parameters for the films.

Sample	Young’s Modulus (MPa)	Elongation at Break (%)	Tensile Strength at Break (MPa)	Tenacity (Nmm/mm^3^) (**)	Elongation Yield (%)	Tensile Yield (MPa)
Bilayer	578 ± 121 (*)	13.9 ± 1.9 (*)	16.7 ± 3.2 (*)	1.74 ± 0.19	3.8 ± 0.6	12.9 ± 2.6
Hydrophobic monolayer	1702 ± 130	65 ± 83	20.6 ± 3.7	13 ± 16	3.2 ± 0.4	34.4 ± 3.7
Hydrophilic monolayer	104 ± 20	16.8 ± 1.4	10.9 ± 1.7	0.88 ± 0.16	-	-
TM Hydrophobic	169 ± 26	490 ± 47	13.8 ± 1.8	43.3 ± 7.1	-	-
TM Hydrophilic	159 ± 24	16.2 ± 0.5	12.7 ± 1.0	1.05 ± 0.1	-	-

(*) Corresponds to the point of delamination rather than complete rupture. (**) As calculated from the area below the stress-strain curve and expressed as stored mechanical energy/sample volume.

**Table 5:** Permeance and permeability for the films.

Sample	Permeance (g/m^2^ d Pa)	Permeability (g m/m^2^ d Pa) × 10^6^
Bilayer	0.0518	6.9
Hydrophobic monolayer	0.0666	2.2
Hydrophilic monolayer	0.7987	85
TM Hydrophobic	0.0074	1.0
TM Hydrophilic	0.7914	100

## Data Availability

Dataset available on request from the authors.
